# The Impact of Changes in Work Arrangements During COVID-19 Pandemic on the Lifestyle of Qatar's Working Population

**DOI:** 10.1097/JOM.0000000000002443

**Published:** 2021-11-23

**Authors:** Muna Abed Alah, Sami Abdeen, Vahe Kehyayan, Iheb Bougmiza

**Affiliations:** Community Medicine Department, Hamad Medical Corporation (HMC) (Dr Abed Alah, Dr Abdeen); University of Calgary in Qatar (Dr Kehyayan); Community Medicine Department, Primary Health Care Corporation (PHCC) (Dr Bougmiza), Doha, Qatar; Community Medicine Department, College of Medicine, Sousse University, Tunisia (Dr Bougmiza).

**Keywords:** COVID-19, diet, lifestyle, physical activity, Qatar, work from home

## Abstract

**Objectives:**

To explore the impact of changing work arrangements during COVID-19 on diet, physical activity, body weight, and sleep of Qatar's working population.

**Methods:**

A web-based survey targeting working adults who were residing in Qatar during the period of home confinement was conducted.

**Results:**

About 47% of 1061 participants reported weight gain. Higher proportions of participants reported consuming more fatty foods (*P* = 0.007), more sugary foods (*P* = 0.001), and greater increase in screen and sitting/reclining times (*P* < 0.001) among the work from home (WFH) group. Participants with higher adverse dietary changes score were more likely to report weight gain in both the WFH (adjusted OR 1.38, 95% CI 1.28 to 1.49), and working regularly groups (adjusted OR, 1.31, 95% CI 1.20 to 1.43) with *P* < 0.001.

**Conclusion:**

Qatar's working population experienced adverse lifestyle changes which were more prominent among those who shifted to WFH.

The COVID-19 pandemic impacted almost every aspect of our work and life with wide ranging health and economic consequences.^1^ Many countries worldwide imposed various restrictions to contain the spread of the COVID-19 infection. Governments and authorities everywhere encouraged physical distancing and home confinement measures, discouraged social gatherings, and closed schools, gyms, and shopping malls.^2^ Many organizations and institutions encouraged their employees to work from home (WFH) in order to stay safe.^3^ Consequently, homes have become the new workplace, school, and gym for many people. Despite the essential benefits brought about by movement restrictions and home confinement measures on containing the spread of COVID-19 infection, adverse health consequences of such restrictions began to evolve. The current evidence has shown how several aspects of lifestyle such as dietary behaviors, physical activity, weight, and sleep are impacted in a health-compromising direction. People are experiencing more unhealthy dietary changes such as frequent snacking, eating larger quantities,^4–8^ less physical activity,^4–11^ an increase in daily sitting and screen times,^5,7,8,11^ weight gain,^6^ and sleep disturbances.^12^ Qatar has implemented swift protective measures to mitigate the spread of COVID-19 beginning March 2020, such as limiting the number of people going to work and switching to WFH. A recent study has shown that working from home during COVID-19 pandemic adversely affected several lifestyle aspects. Of those who shifted to WFH, 39% reported eating larger quantities of food, 33% reduction in daily physical activity, 41% weight gain, and 21% reported insomnia.^13^ Decreased walking among those worked from home during the pandemic was observed in another study and was linked to depression.^14^ To our knowledge, only few studies have assessed the impact of working from home on lifestyle aspects during COVID-19. At the time of conducting this study, most of the available literature on WFH lacked contextual relevance in the current pandemic and only few studies conducted in Qatar investigated the implications of WFH during COVID-19 pandemic and its impact on several lifestyle aspects. This unprecedented crisis of COVID-19 is an opportunity to explore the health effects brought about by changing normal work life from typical workplaces to WFH. In this study we aimed to assess the impact of working from home as part of COVID-19 related home confinement measures on several lifestyle aspects including dietary behaviors, physical activity, body weight and sleep in Qatar. Our objectives were to explore the impact of changes in work arrangements during COVID-19 pandemic on several lifestyle aspects as reported by Qatar's working population and compare the changes between those who had shifted to WFH during COVID-19 related home confinement measures and those who had continued working regularly. We believe that the results of this study will guide the implementation of effective lifestyle related interventions targeting Qatar's working population.

## METHODS

### Study Design and Target Population

A cross-sectional study was conducted between January and February 2021. The target population included working adults more than or equal to 18 years regardless of type of profession or occupation who were residing in Qatar during the period of COVID-19 related home confinement measures.

### Study Procedure

Data were collected through an online, anonymous self-administered questionnaire developed using SurveyMonkey software (SurveyMonkey Inc, San Mateo, CA). Data were collected between January 4, 2021 and February 28, 2021. Participants were recruited through social media platforms of the Hamad Medical Corporation (HMC) that are generally accessible by the public. In addition, snowball sampling was used to extend the recruitment by circulating the link through emails and WhatsApp groups. Reminders with reposting of the links on social media were done on a regular basis. Ethical approval was obtained from the Institutional Review Board (IRB) of Hamad Medical Corporation.

### Study Questionnaire

The questionnaire was adopted from other validated and reliable questionnaires.^15,16^ It was developed initially in English, then translated into three other languages (Arabic, Malayalam, and Urdu) by an accredited translation body. We examined the face and content validities of the questionnaire. To examine the face validity, experts in the field (lifestyle medicine and community medicine specialists) evaluated the questionnaire for feasibility, readability, consistency of style, formatting, and clarity of the language used. They were requested to propose a better formulation, and their comments were discussed by the research team until consensus was reached on the items to be included in the final version of the questionnaire. To examine content validity, the items were evaluated for relevance and rated as: 1 not relevant, 2 somewhat relevant, 3 quite relevant, and 4 highly relevant. Their evaluation results were evaluated by calculating content validity index and showed satisfactory content validity. It consisted of four sections. The first section explored the sociodemographic characteristics of participants (age, gender, nationality, marital status, educational level, nature of work, whether participants shifted to work from home or not during home confinement measures, and personal history of chronic diseases). The second and third sections explored the changes in dietary behaviors, and changes in physical activity (including changes in time spent in exercise, sitting/reclining, and screen time) and body weight, respectively. The last section assessed changes in sleep including sleep duration and quality.

### Outcome Measures

We measured the perceived changes in several lifestyle aspects as reported by the participants and compared the changes between those who worked from home and those who continued working regularly in their usual workplace. Changes in diet were assessed by first, asking participants to report their overall perception of dietary habits as becoming healthier, less healthy, or stayed the same. Second, we asked participants to indicate their degree of agreement on a four-point Likert scale with seven statements (five indicating unhealthy changes, and two indicating healthy changes). The points on the scale are 1 (strongly disagree), 2 (disagree), 3 (agree), 4 (strongly agree). Examples of statements include: “I tend to eat more fatty food,” “I tend to eat more sugar/chocolate/sweets,” “I tend to eat more junk food,” “I tend to eat more vegetables/fruits.” We calculated an adverse dietary changes score by summing the scores for each of the five unhealthy change statements with a score ranging from 4 to 20 points. The underlying causes for dietary changes were assessed for each direction of change. To assess the changes in physical activity and sedentary behaviors, participants were asked to report the average time spent in front of screens (screen time), sitting/reclining and exercise, expressed as hours/day before and during home confinement measures. The perceived changes in weight were assessed on an ordinal scale by asking participants to report their average weight gain (no change, less than 3 kg, 3 to 6 kg, 7 to 10 kg, more than 10 kg). With regard to sleep, we asked participants to report the average hours of sleep per day before and during home confinement, to rate their overall subjective sleep quality on a five-point Likert scale as 1 (very good), 2 (good), 3 (average), 4 (poor), and 5 (very poor), and to indicate their degree of agreement on six statements assessing sleep latency, disturbances, and daytime dysfunction using a four-point Likert scale: 1 (strongly disagree), 2 (disagree), 3 (agree), and 4 (strongly agree). We also calculated an adverse sleep quality score by summing the scores for each of the six statements with scores ranging from 6 to 24 points.

### Statistical Analysis

We analyzed the data using the IBM SPSS Statistics for Windows, version 26 (IBM Corp., Armonk, NY). Descriptive statistics were presented as frequencies and percentages for categorical variables. We tested the normality of continuous variables using Shapiro-Wilk test. The nonparametric Mann–Whitney *U* test was used to compare ordinal and not normally distributed continuous variables between those who worked from home and those who worked at their usual place of employment. The Wilcoxon Signed Rank test was used to test the differences in screen, sitting/reclining, and exercise times, and sleep duration and quality as expressed before and during the home confinement measures. We calculated the effect size for these comparisons using Rank biserial correlation (small 0.10 to less than 0.30, medium 0.30 to less than 0.50, large more than or equal to 0.50). Multivariable logistic regression was executed to determine predictors of weight gain among those who worked from home and those who continued working regularly at their workplaces. Hosmer and Lemeshow test was used to assess goodness of fit. The associations between risk factors and outcomes are presented as adjusted odds ratios (ORs) and 95% confidence intervals (CIs). *P*-values of <0.05 were considered significant for all statistical tests.

## RESULTS

### Sociodemographic Characteristics

The questionnaire was completed by 1061 participants. Majority were men (757; 71.3%), 35 to 54 years old (585; 55.1%), married (850; 80.1%), and have completed college or a higher degree of education (832; 78.4%). Over 50 nationalities were reported by participants with the most common being Indian nationality (56.6%). Only 37 (3.5%) Qatari nationals participated. Of the total participants, 565 (53.3%) shifted to WFH since the start of the home confinement measures, while the remaining 496 (46.7%) continued to work regularly at their usual workplaces. Of those who shifted to WFH, 468 (82.8%) reported that their usual job involved mostly office work compared to 54.6% of those who continued to work regularly at their usual place of work. Table 1 describes the sociodemographic characteristics of the participants by working status.

**TABLE 1:** Participants’ Background Characteristics

Variable	Work From Home Group (*n* = 565) No (%)	Working Regularly Group (*n* = 496) No (%)
Age		
18–34	209 (37.0)	220 (44.4)
35–54	332 (58.8)	253 (51.0)
55+	24 (4.2)	23 (4.6)
Gender		
Male	360 (63.7)	397 (80.0)
Female	205 (36.3)	99 (20.0)
Nationality (classified by regions)^∗^		
Americas	24 (4.2)	4 (0.8)
Sub-Saharan Africa	19 (3.4)	21 (4.2)
Europe	64 (11.3)	14 (2.8)
Middle East - North Africa	99 (17.5)	87 (17.5)
Asia - Pacific	359 (63.5)	370 (74.6)
Highest degree of education		
No formal education	4 (0.7)	10 (2.0)
High school diploma	65 (11.5)	102 (20.6)
College or higher	478 (84.6)	354 (71.4)
Vocational training	18 (3.2)	30 (6.0)
Nature of work		
Mostly office work	468 (82.8)	271 (54.6)
Mostly field work	97 (17.2)	225 (45.4)
Marital status		
Married	449 (79.5)	401 (80.8)
Not married	116 (20.5)	95 (19.2)
Presence of chronic disease/s^†^		
Yes	136 (24.1)	108 (21.8)
No	429 (75.9)	388 (78.2)

∗ More than 50 different nationalities were reported.

† Most commonly reported chronic diseases for both populations were: diabetes, hypertension, asthma, and cardiovascular diseases respectively.

### Dietary Changes

Of all participants, 359 (33.8%) perceived an overall healthier dietary change, and 279 (26.3%) perceived an overall unhealthy change. Of those who shifted to WFH, 212 (37.5%), and 169 (29.9%) perceived a healthier, and unhealthy dietary changes respectively, while of those who continued to work regularly 147 (29.6%) and 110 (22.2%) reported healthier and unhealthy changes respectively. No significant changes were found between the WFH and working regularly groups in the overall perception of diet. Significantly higher proportions of participants reported consuming more fatty foods (*P* = 0.007) and more sugary foods and sweets (*P* = 0.001) among those who worked from home compared with those who did not. At the same time, significantly higher proportions of participants reported more dependance on home cooking since the start of home confinement measures among those who worked from home compared with those who did not (*P* = .044). No significant difference was found between the 2 groups in the total adverse dietary changes score (Table 2). The most common reported causes for unhealthy dietary changes among both groups were similar and included feeling of boredom, longer time spent in front of screens, and increasing feelings of stress during the pandemic leading to consuming larger food quantities and more frequent snacking during home confinement measures. Concerning the underlying causes for healthier changes, both groups also reported similar causes including participants’ beliefs that healthier eating strengthens immunity and fear of contracting COVID-19 infection if they ordered food from outside.

**TABLE 2:** Differences in Dietary and Sleep Quality Related Behaviors Between Work From Home and Working Regularly Groups During COVID-19 Related Home Confinement Measures

	Work From Home Group (*n* = 565)	Working Regularly Group (*n* = 496)	
Statement	Mean Ranks	Mean Ranks	*P*-Value^∗^
Unhealthy dietary statements			
I tend to eat more fatty food	553.2	505.7	0.007
I tend to eat more sugar/chocolate/sweets	558.7	494.5	**0.001**
I tend to eat more fast/junk food	539.8	521.0	0.275
I tend to eat more processed/canned food than fresh food	520.9	542.5	0.206
I tend to eat larger quantities of food	539.1	571.8	0.321
Adverse dietary changes score^†^	548.0	511.6	0.52
Healthy dietary behaviors			
I tend to eat more vegetables and/or fruits	518.5	545.3	0.114
I tend to depend more on home cooking	547.3	512.4	**0.044**
Adverse sleep statements			
My sleeping pattern has changed (I tend to sleep more during daytime than nighttime).	517.9	545.9	0.113
It is difficult for me to fall asleep at night	528.5	533.8	0.764
It is difficult for me to stay asleep all night	530.5	531.6	0.947
It is difficult for me to go to sleep again if I woke up at night	537.8	523.3	0.407
I often wake up from sleep still feeling tired	543.6	516.7	0.123
I often have trouble staying awake while driving, eating meals, or engaging in social activity	504.8	560.8	**0.001**
Adverse sleep change score^‡^	529.2	533.1	0.832

∗ Using Mann–Whitney *U* test. *P* values of <0.05 were considered significant.

† Calculated as the sum of the scores of unhealthy dietary changes statements.

‡ Calculated as the sum of the scores of adverse sleep changes statements.

### Changes in Physical Activity and Sedentary Behaviors

Participants among the WFH group reported a significant increase in screen time (2.25 h/d mean increase, 95% CI: 1.99 to 2.51) with *P* < 0.001, a significant increase in sitting/reclining time (2.25 h/d mean increase, 95% CI: 1.93 to 2.57) with *P* < 0.001, and a non-statistically significant decrease in exercise time since the start of home confinement measures. For those who continued working regularly, a significant increase was found in screen time (1.48 h/d mean increase, 95% CI: 1.23 to 1.74) and *P* < 0.001, in sitting/reclining time (1.41 h/d mean increase, 95% CI: 1.13 to 1.70) and *P* < 0.001, while a significant decrease in exercise time (0.11 h/d mean reduction, 95% CI: –0.21 to –0.02) and *P* = 0.007 (supplemental Table). Comparing the differences in the above-mentioned times (before and during home confinement measures) between the WFH and working regularly groups, higher proportions of participants reported greater increase in screen and sitting/reclining times among the WFH group (*P* < 0.001). However, no significant difference was found with regard to exercise time between the groups (Table 3).

**TABLE 3:** Differences in Multiple Lifestyle Aspects (Presented as a Difference Between Before and During Home Confinement Measures) Between the Work from Home and Working Regularly Groups

	Work From Home Group (*n* = 565)	Working Regularly Group (*n* = 496)	
Variable	Mean Ranks	Mean Ranks	*P*-Value^∗^
Screen time difference (h/d)	582.1	472.8	**<0.001**
Sitting/reclining time difference (h/d)	586.5	474.7	**<0.001**
Exercise time difference (h/d)	536.0	525.4	0.537
Sleep duration difference (h/d)	560.3	497.6	**0.001**
Sleep quality score difference	511.9	552.8	**0.019**

∗ Using Mann–Whitney *U* test, *P* values of <0.05 were considered significant.

### Changes in Weight

**FIGURE 1:**
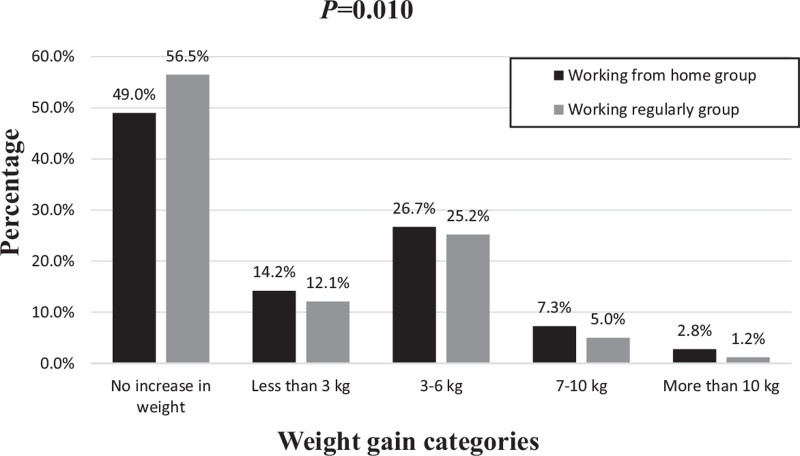
Amount of weight gain reported by the participants during COVID-19 related home confinement measures in the work from home and working regularly groups.

Of the total sample, 504 (47.5%) reported some weight gain since the start of home confinement measures. About half of the participants among the WFH group (288; 51%) reported weight gain, with more than half of them (151; 52.4%) reporting a 3 to 6 kg average weight gain. On the other hand, 216 (43.5%) of the participants who continued to work regularly reported weight gain. Higher proportions of participants reporting higher weight gain categories were found among the WFH group compared with the working regularly group (*P* = 0.010). See Fig. 1.

### Predictors of Weight Gain Among the Participants

As shown in Table 4, two logistic regression models were carried out to determine the predictors of reporting weight gain among the groups. One for the WFH group, and the other for the working regularly group. Both models were of good fit and statistically significant with (*χ*^2^_(24)_ 175.041, *P* < 0.001) and (*χ*^*2*^_(21)_ 140.972, *P* < 0.001) for the first and second models respectively. Approximately 35% and 33% (Nagelkerke *R*^2^) of the variance in the weight gain can be explained by the first and the second models respectively. In the first model, screen time, exercise time, and adverse dietary changes were found significantly and independently associated with reporting weight gain among the WFH group. Participants who did not report a change in screen time were less likely to report weight gain compared with those who increased their screen time (adjusted OR 0.49, 95% CI 0.27 to 0.90, *P* = 0.021). Those who increased their exercise time were less likely to report weight gain compared with those who decreased their exercise time (adjusted OR 0.31, 95% CI 0.17 to 0.54, *P* < 0.001). On the other hand, participants with higher adverse dietary changes score were more likely to report weight gain compared with those with lower scores (adjusted OR 1.38, 95% CI 1.28 to 1.49, *P* < 0.001). In the second model, age, gender, and adverse dietary changes score were significantly and independently associated with reporting weight gain among the working regularly group. Participants aged 35 to 54 (adjusted OR 0.60, 95% CI 0.38 to 0.95, *P* = 0.030), and men (adjusted OR 0.41, 95% CI 0.23 to 0.72, *P* = 0.002) were less likely to report weight gain compared with younger, and female participants, respectively. Similar to the WFH group, the higher the adverse dietary changes score, the more likely participants report weight gain (adjusted OR, 1.31, 95% CI 1.20 to 1.43, *P* < 0.001).

**TABLE 4:** Predictors of Weight Gain Among the Work From Home and Working Regularly Groups Using Multivariable Logistic Regression Analysis

	Work From Home Group (*n* = 565)			Working Regularly Group (*n* = 496)		
	Reported Weight Gain	AOR (95% CI)	*P*-Value	Reported Weight Gain	AOR (95% CI)	*P*-Value
Variable	No (%)			No (%)		
Age						
18–34	121 (57.9)	1 [Reference]		116 (52.7)	1 [Reference]	
35–54	158 (57.6)	1.04 (0.67–1.62)	0.853	94 (37.2)	0.60 (0.38–0.95)	0.030
55+	9 (37.5)	0.96 (0.33–2.81)	0.936	6 (26.1)	0.39 (0.12–1.27)	0.117
Gender						
Male	167 (46.4)	0.78 (0.50–1.24)	0.294	156 (39.3)	0.41 (0.23–0.72)	0.002
Female	121 (59.0)	1 [Reference]		60 (60.6)	1 [Reference]	
Nationality (classification by regions)						
Americas	12 (50.0)	0.78 (0.27–2.23)	0.637	3 (75.0)	1.96 (0.15–26.07)	0.609
Sub-Saharan Africa	13 (58.4)	1.98 (0.60–6.47)	0.260	12 (57.1)	0.95 (0.32–2.80)	0.927
Europe	40 (62.5)	1.37 (0.67–2.81)	0.384	10 (71.4)	1.40 (0.31–6.24)	0.659
Middle East - North Africa	57 (57.6)	1.07 (0.62–1.86)	0.807	48 (55.2)	0.90 (0.51–1.60)	0.714
Asia - Pacific	166 (46.2)	1 [Reference]		143 (38.6)	1 [Reference]	
Highest degree of education						
No formal education	1 (25.0)	1 [Reference]		3 (30.0)	1 [Reference]	
High school diploma	27 (41.5)	2.62 (0.127–53.85)	0.532	30 (29.4)	1.84 (0.37–9.01)	0.455
College or Higher	250 (52.3)	3.63 (0.182–72.45)	0.399	169 (47.7)	1.88 (0.40–8.83)	0.426
Vocational training	10 (55.6)	3.72 (0.15–91.39)	0.421	14 (46.7)	2.81 (0.50–15.68)	0.240
Nature of work						
Mostly office work	242 (51.7)	1.32 (0.76–2.29)	0.328	136 (50.2)	1.49 (0.96–2.33)	0.079
Mostly field work (out of office work)	46 (47.4)	1 [Reference]		80 (35.6)	1 [Reference]	
Duration of working from home						
Less than 1 month	34 (41.0)	1 [Reference]		----------	----------	-------
1–2 months	58 (53.2)	1.33 (0.67–2.63)	0.41	----------	----------	-------
3–4 months	88 (53.0)	1.37 (0.73–2.56)	0.32	----------	----------	-------
5 months or more	108 (52.2)	1.36 (0.74–2.50	0.32	----------	----------	-------
Marital status						
Married	222 (49.4)	1.12 (0.66–1.90)	0.67	163 (40.6)	0.95 (0.54–1.66)	0.853
Not married	66 (56.9)	1 [Reference]		53 (55.8)	1 [Reference]	
Chronic disease						
Yes	57 (41.9)	0.702 (0.43–1.14)	0.149	41 (38.0)	0.99 (0.52–1.54)	0.687
No	231 (53.8)	1 [Reference]		175 (54.1)	1 [Reference]	
Attending gyms regularly before home confinement measures						
Yes	81 (61.8)	1.25 (0.75–2.09)	0.386	62 (63.9)	1.71 (0.96–3.02)	0.067
No	207 (57.7)	1 [Reference]		154 (38.6)	1 [Reference]	
Screen time						
Decreased	29 (50.9)	1.30 (0.60–2.82)	0.505	18 (36.7)	0.69 (0.32–1.51)	0.355
No change	32 (30.5)	0.49 (0.27–0.90)	**0.021**	56 (29.0)	0.59 (0.33–1.06)	0.077
Increase	227 (56.3)	1 [Reference]		142 (55.9)	1 [Reference]	
Sitting time						
Decreased	28 (43.8)	0.72 (0.35–1.47)	0.366	23 (37.1)	0.74 (0.36–1.54)	0.423
No change	48 (38.4)	1.13 (0.64–1.99)	0.686	62 (31.0)	0.56 (0.32–1.00)	0.051
Increase	212 (56.4)	1 [Reference]		131 (56.0)	1 [Reference]	
Exercise time						
Decreased	118 (74.2)	1 [Reference]		74 (66.1)	1 [Reference]	
No change	122 (44.9)	0.47 (0.28–0.78)	**0.003**	114 (37.3)	0.71 (0.41–1.23)	0.217
Increase	48 (53.8)	0.31 (0.17–0.54)	**<0.001**	28 (35.9)	0.60 (0.30–1.21)	0.152
Adverse dietary changes score^∗^	----------	1.38 (1.28–1.49)	**<0.001**	----------	1.31 (1.20–1.43)	**<0.001**

AOR, adjusted odds ratio; CI, confidence interval.

∗ Adverse dietary changes score means for those who reported weight gain and those who reported no weight gain in the work from home group were (13.1, 10.2) respectively while in the working regularly group were (12.6, 10.3) respectively .

### Changes in Sleep

Out of all participants, 488 (46%) reported an increase in sleep duration, 149 (14%) reported a decrease, and the remaining reported no change in duration since the start of home confinement measures. In the WFH group, the mean sleep duration increased significantly from 6.90 h/d before to 7.78 h/d during home confinement (0.89 hour mean difference, 95% CI: 0.74 to 1.04, *P* < 0.001). While in the working regularly group the mean duration increased significantly from 6.93 to 7.55 h/d (0.63 hour mean difference, 95% CI: 0.47 to 0.78, *P* < 0.001). Upon comparing the difference in sleep duration (before and during home confinement) between the WFH and working regularly groups, we found a significantly higher proportions of participants reporting greater increase in sleep duration among the WFH group (*P* = 0.001). Concerning the overall subjective sleep quality, 165 (29.2%), 131 (26.4%) of those who worked from home, and those who continued working regularly reported poorer sleep quality during home confinement as compared with before respectively. Higher proportions of participants reported trouble staying awake while driving, eating meals, or engaging in social activity among the working regularly group compared with the WFH group (*P* = 0.001). However, no significant difference was found between the groups in the total adverse sleep quality score (Table 2).

## DISCUSSION

Movement restriction and COVID-19 related home confinement measures recommended by countries worldwide resulted in working from home becoming a policy priority for most governments including Qatar. Despite the overall favorability of many workers for working from home, it can affect their health and adversely impact their lifestyle. In this study, we explored the impact of working from home as part of COVID-19 related home confinement measures on several lifestyle aspects including dietary behaviors, physical activity, body weight, and sleep. In addition, we compared lifestyle changes reported by workers between those who shifted to WFH and those who continued working regularly at their usual place of work.

### Dietary Changes

Concerning dietary changes, higher proportions of participants in the WFH group reported consuming more fat rich, and sugary food compared with those who continued working regularly. This might be explained by the fact that those working from home have an easier access to such unhealthy food choices, compared with those working at their workplaces because many companies and institutions in Qatar follow the recommendations of the Ministry of Public Health to provide healthy foods in canteens for their employees.^17^ Also, high levels of stress experienced by people during home confinement especially those who WFH,^18^ might force them toward consuming more comfort foods which in most cases include fatty and sugary items. For example, with closure of schools, children were forced to stay home,^19,20^ serving as a source of distraction for their working parents at home especially with the lack of support from day care centers or babysitters during working hours in view of the pandemic. Also, many parents had to deal with home schooling of their children and those who worked from home were more likely to deal with this than those who continued working regularly.^21–23^ These overlapping responsibilities may lead to building up stress.^22,23^ In addition, for others, blurred work-life boundaries with diminished boundaries between one's work and family life may make it difficult to detach psychologically and mentally from work when at home which may further increase stress.^24^ On the other hand, participants from the WFH groups were more likely to report more dependence on home cooking than the other group. This finding is expected since those working from home have more time to cook than those working regularly.

### Changes in Physical Activity and Sedentary Behaviors

The availability of time for the WFH group might also be a reason behind the significantly higher proportions of participants reporting greater increases in screen and sitting/reclining times compared with the other group as they may use the extra time they have laying down relaxing, watching TV, playing video games, and using social media platforms.^25^ Another explanation may be that working remotely requires the utilization of different telecommunication methods resulting in increased screen time.^26^ In addition, having around one-fifth of participants among the WFH group originally working in field work (out of office) prior to the home confinement measures and then suddenly shifting to work from home might explain the higher increases in sitting/reclining time among them.

### Changes in Body Weight and Predictors of Weight Gain

Almost one half (47%) of the participants in the total sample reported some weight gain consisting with the results of a study conducted in Bangkok which showed that 41% of workers who shifted to work from home reported weight gain.^13^ This finding is alarming and might result in complications of existing chronic diseases and can contribute to the development of new ones. There was a significant difference between the WFH group, and the working regularly group in terms of weight gain, where unsurprisingly greater weight gain was reported among the WFH group. The higher the numbers of adverse dietary behavioral changes adopted as reflected by the adverse dietary changes score, the higher was the probability of reporting weight gain supporting the finding of a previous study.^27^ Unsurprisingly, we found that not increasing the screen time, and increasing exercise time is protecting against weight gain among the WFH group supporting the available literature.^28^ Men were less likely than women to report weight gain among the working regularly group. One explanation might be the higher level of stress women experienced during this pandemic compared with men as evident in the literature,^29^ which can trigger emotional eating leading to weight gain^30^

### Changes in Sleep

Concerning sleep, the WFH group had relatively more participants reporting greater increases in average sleep duration/day since the start of home confinement measures and shifting to WFH. Being able to work from home, near their bedrooms, at their own convenience, and not being supervised by other employees or by their employers might explain this finding.

This study has shown that home confinement measures exerted some adverse health effects on several lifestyle aspects of the working population in Qatar. These effects were generally more prominent in the WFH group compared with those who continued working regularly. The sudden shift to WFH for long periods of time could not have been anticipated by workers, so we assumed it may have been difficult for them to adapt to the “new normal” of working remotely which had an adverse impact on their health-related behaviors. Additionally, such a sudden shift in the work setting in the context of stressful circumstances linked to the pandemic might further contribute to the problem. Evidence has shown that the use of social media by organizations to promote a healthy lifestyle among its employees can constitute an innovative and promising intervention.^31^ We believe that it is now the right time to invest in such interventions, as many organizations are depending on tele-communication and social media platforms as ways of communication with employees while working remotely. Employers and organizations can help promoting healthy lifestyles of their employees by giving them free access to health clubs, personal training, food logs, cookbooks and healthy eating supplies, and by implementing environmental dietary modification such as menu modification, healthy food price discounts, strategic positioning of healthier alternatives, and portion size control at the workplaces.^32,33^ Moreover, providing awareness sessions and educating employees on planning their snack times and identifying stress-relieving activities that do not involve food may help them manage weight gain.^28^

### Strengths and Limitations

We believe that this study had several strengths. We were able to recruit an acceptable sample size of 1061 participants. Luckily, we had comparable percentages of those who shifted to WFH and those who did not which enabled us to compare the results between them. This study is one of the few conducted worldwide and particularly in the Middle East to address the impact of WFH on several lifestyle aspects in the context of COVID-19 pandemic. Additionally, having the questionnaire available for participants in four languages can be considered a point of strength in a multilingual country like Qatar.

We also acknowledge that this study had a few limitations. Firstly, our sampling technique and snowballing might have introduced selection bias and affected the representativeness of the sample. Secondly, the data and measurements obtained (such as screen, sitting/reclining, and exercise times, amount of weight gain, and sleep duration) were self-reported and liable to information bias. Additionally, in many of the questions, we asked participants to compare the changes during and before home confinement measures which might have resulted in recall bias. However, using web-based self-reported surveys are currently the safest means of addressing different research areas in view of physical distancing measures recommended during the pandemic. Thirdly, we only ensured face and content validity for the translated questionnaires and did not assess their reliability. Lastly, we built our conclusion about changes in physical activity mainly on the reduction in exercise times and the increase in sedentary behaviors. However, other forms of physical activities such as house chores that we did not assess might have increased. So physical activity related results should be interpreted with caution.

## CONCLUSION

The results of this study indicate that Qatar's working population experienced adverse changes in different lifestyle aspects including diet, physical activity, and sleep since the start of COVID-19 related home confinement measures. These changes were more prominent among those who shifted to WFH compared with those who did not. Employers, organizations, health care workers, and governments have the responsibility of encouraging those working from home to maintain healthy lifestyle behaviors. WFH practice might become the “new normal” or it might be imposed during any public health crises or any potential future outbreaks. Thus, organizations need to adapt their polices and rules to fit this new work environment in a way that ensures the health, wellbeing, and safety of their employees. More efforts need to be directed toward the implementation of effective lifestyle related interventions tailored to those working from home. Future research needs to focus on investigating the persistence of adverse lifestyle changes in the post pandemic era and on actual measurements of body weight, exercise time, sleep, and time spent in sedentary behaviors instead of relying solely on self-reporting by participants.

## Supplementary Material

Supplemental Digital Content
