# Supplementary material for: The Impact of Changes in Work Arrangements During COVID-19 Pandemic on the Lifestyle of Qatar's Working Population
Source: J Occup Environ Med. 2021 Nov 23;64(2):e53–9. doi: 10.1097/JOM.0000000000002443 (PMC8808759; doi:10.1097/JOM.0000000000002443)
Supplement: Supplemental Digital Content [file joem-64-e53-s001.docx]

Supplemental Table: Changes in screen, sitting/reclining, and exercise times, and sleep duration before and during COVID-19 related home confinement measures in the total sample, and in each of the work from home and working regularly groups.

| **Variable** | | **Work from home group** | | | **Working regularly**  **group** | | | **Total Sample** | | |
| --- | --- | --- | --- | --- | --- | --- | --- | --- | --- | --- |
|  |  | **Median (IQR)** | ***P*-value*** | ***r*** | **Median (IQR)** | ***P*-value*** | ***r*** | **Median (IQR)** | ***P*-value*** | ***r*** |
| **Screen Time (hours/day)** | Before | 4 (2-8) | **>0.001** | 0.79 | 3 (2-5) | **>0.001** | 0.75 | 3 (2-6) | **>0.001** | 0.77 |
|  | During | 7 (4-10) |  |  | 5 (2-8) |  |  | 6 (3-9) |  |  |
| **Sitting/Reclining Time (hours/day)** | Before | 4 (2-8) | **>0.001** | 0.72 | 3 (2-5.5) | **>0.001** | 0.69 | 4 (2-7) | **>0.001** | 0.71 |
|  | During | 7 (4-10) |  |  | 4 (2-8) |  |  | 6 (3-10) |  |  |
| **Exercise Time (hours/day)** | Before | 1 (1-1) | 0.208 |  | 1 (1-1) | **0.007** | -0.22 | 1 (1-1) | **0.007** | -0.13 |
|  | During | 1 (0-1) |  |  | 1 (0-2) |  |  | 1 (0-2) |  |  |
| **Sleep Duration (hours/day)** | Before | 7 (6-8) | **>0.001** | 0.60 | 7 (6-8) | **>0.001** | 0.54 | 7 (6-8) | **>0.001** | 0.58 |
|  | During | 8 (7-9) |  |  | 7 (6-8) |  |  | 8 (7-9) |  |  |

Abbreviations: IQR, Interquartile range; *r*, Rank-biserial correlation coefficient

* Using Wilcoxon signed rank test.
